# Current methods for the detection of *Plasmodium* parasite species infecting humans

**DOI:** 10.1016/j.crpvbd.2022.100086

**Published:** 2022-03-19

**Authors:** Lucinda Slater, Shoaib Ashraf, Osama Zahid, Qasim Ali, Muhammad Oneeb, Muhammad Haroon Akbar, Muhammad Ilyas Riaz, Kiran Afshan, Neil Sargison, Umer Chaudhry

**Affiliations:** a Royal (Dick) School of Veterinary Studies, University of Edinburgh, UK; b Massachusetts General Hospital and Harvard Medical School, Boston, MA, USA; c University of Veterinary and Animal Sciences Lahore, Pakistan; d Quaid-i-Azam University, Islamabad, 45320, Pakistan; e School of Veterinary Medicine, University of Surrey, UK; f Univeristy of Agriculture D. I. Khan, Pakistan

**Keywords:** Malaria, Diagnosis, *Plasmodium* species, Identification

## Abstract

Malaria is the world’s fatal parasitic disease. The ability to quickly and accurately identify malaria infection in challenging environments is crucial to allow efficient administration of the best treatment regime for human patients. If those techniques are accessible and efficient, global detection of *Plasmodium* species will become more sensitive, allowing faster and more precise action to be taken for disease control strategies. Recent advances in technology have enhanced our ability to diagnose different species of *Plasmodium* parasites with greater sensitivity and specificity. This literature review provides a summary and discussion of the current methods for the diagnosis and identification of *Plasmodium* spp. in human blood samples. So far not a single method is precise, but advanced technologies give consistent identification of a *Plasmodium* infection in endemic regions. By using the power of the recent methods, we can provide a broader understanding of the multiplicity of infection and or transmission dynamics of *Plasmodium* spp. This will result in improved disease control strategies, better-informed policy, and effective treatment for malaria-positive patients.

## Highlights

• Summary of the methods currently available for the detection of *Plasmodium* spp. infecting humans.
• No single method is perfect for every application to identify *Plasmodium* spp.
• Newly developed methods give promise for more reliable characterisation of *Plasmodium* spp.

## Graphical abstract

**Figure:**
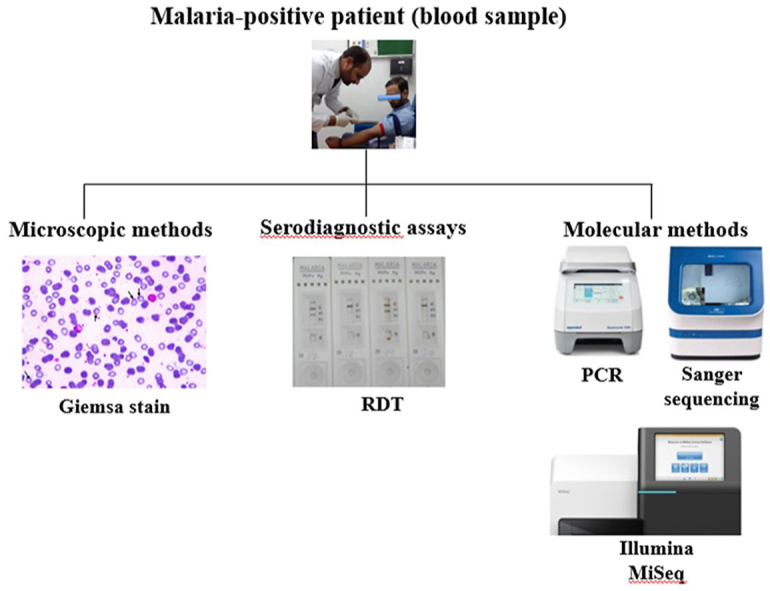

## 1 Introduction

Malaria is responsible for an estimated 228 million cases and 405,000 deaths worldwide in the survey of 2018 (WHO, 2019). In endemic areas, the burden of the illness is immense, despite the development of partial immunity among these populations. Malaria was responsible for the loss of an estimated 45,000 disability-adjusted life years (DALYs) in 2017 alone (Kyu et al., 2018). The uncomplicated disease causes fever, chills, headaches, muscle aches, nausea and vomiting. The more severe disease often develops among vulnerable groups, including children under five, immunocompromised individuals such as pregnant women or patients with acquired immune deficiency syndrome (AIDS), and individuals with no pre-existing immunity, including tourists and migrants. A number of these cases develop cerebral malaria, causing coma, seizures, metabolic acidosis, and death (Autino et al., 2012). The aim of the present review is to summarise the methods currently available for the detection of *Plasmodium* spp. infecting humans. There is no single method being perfect for every application to identify *Plasmodium*. Therefore new methods should promise for more reliable and efficient characterisation of *Plasmodium* spp.

Malaria in humans is caused by the four most common *Plasmodium* species, i.e. *Plasmodium falciparum*, *Plasmodium malariae*, *Plasmodium ovale* (subspecies *P. o. cutisi* and *P. o. wallikeri*), *Plasmodium vivax*, and four zoonotic species, i.e. *Plasmodium brasilianum*, *Plasmodium cynomolgi*, *Plasmodium knowlesi* and *Plasmodium inui*. The most severe infection and the highest death rates are caused by *P. falciparum*, widespread across the malaria-endemic regions across sub-Saharan Africa, Southeast Asia, South America and Western Pacific. *Plasmodium vivax* prevails over *P. falciparum* in South America and both species are common in Southeast Asia (Autino et al., 2012). Although the distribution of *P. malariae* is reported as being patchy, it is spread in all major malaria-endemic regions of the world, with a distribution overlapping that of *P. falciparum*. The highest transmission of *P. malariae* is found across sub-Saharan Africa and parts of Oceania such as Papua and New Guinea. *Plasmodium ovale* was thought to have a much more limited distribution, with endemic transmission traditionally described as being limited to Africa, eastern parts of Indonesia and the Philippines and the Middle East (Mueller et al., 2007). The distribution of *P. knowlesi* is limited to cetrain forested areas in Southeast Asia*. Plasmodium cynomolgi* and *P. inui* were shown to be found naturally throughout Southeast Asia including Sri Lanka, Malaysia, Cambodia and Taiwan in various macaque and Old World monkeys. *Plasmodium brasilianum* is a quartan malaria parasite of New World monkeys in South America (Lalremruata et al., 2015).

To treat a patient showing symptoms of malaria, it is important to diagnose *Plasmodium* spp. infection to rule out other possible causes, and to give them the best chance of recovery. Symptoms such as fever, headaches, chills, nausea and general malaise can be attributed to many different illnesses, so clinical diagnosis alone is not always reliable. Furthermore, many early malaria symptoms are nonspecific and similar to several other diseases including babesiosis (Amexo et al., 2004). Clinical diagnosis does not allow the identification of the species of *Plasmodium* causing infection, which has implications for treatment and is therefore important in cases where a patient may have been exposed to multiple species of *Plasmodium* (Tangpukdee et al., 2009). In many malaria-endemic countries, co-infections with *Plasmodium* spp. are present. In these regions, it is therefore critical to be able to speciate malaria infection to determine which drug has a low risk of encountering resistance and has the best chance of providing effective treatment for the particular infection. In addition, identification of the species of *Plasmodium* infecting malaria patients is also important for disease epidemiology and surveillance, to allow data collection and observation of any trends in a particular area (Erdman & Kain, 2008).

The risk of misdiagnosis can be minimised by using a combination of clinical and laboratory diagnostic methods. The laboratory diagnostics range from traditional microscopy to more complex molecular techniques for the detection of antibodies or parasite nucleic acids (Tangpukdee et al., 2009). While molecular techniques require more advanced equipment and specialist training, many are also capable of detecting which species of *Plasmodium* is present in a sample. The diagnosis of malaria using laboratory methods can be done in several ways. Some of these methods intrinsically allow differentiation between *Plasmodium* spp. due to their recognition of species-specific genetic markers or allow identification to be carried out alongside diagnosis. Each method has its own benefits and drawbacks, which make it better suited to particular applications.

## 2 *Plasmodium* spp. life-cycle

**Fig. 1:**
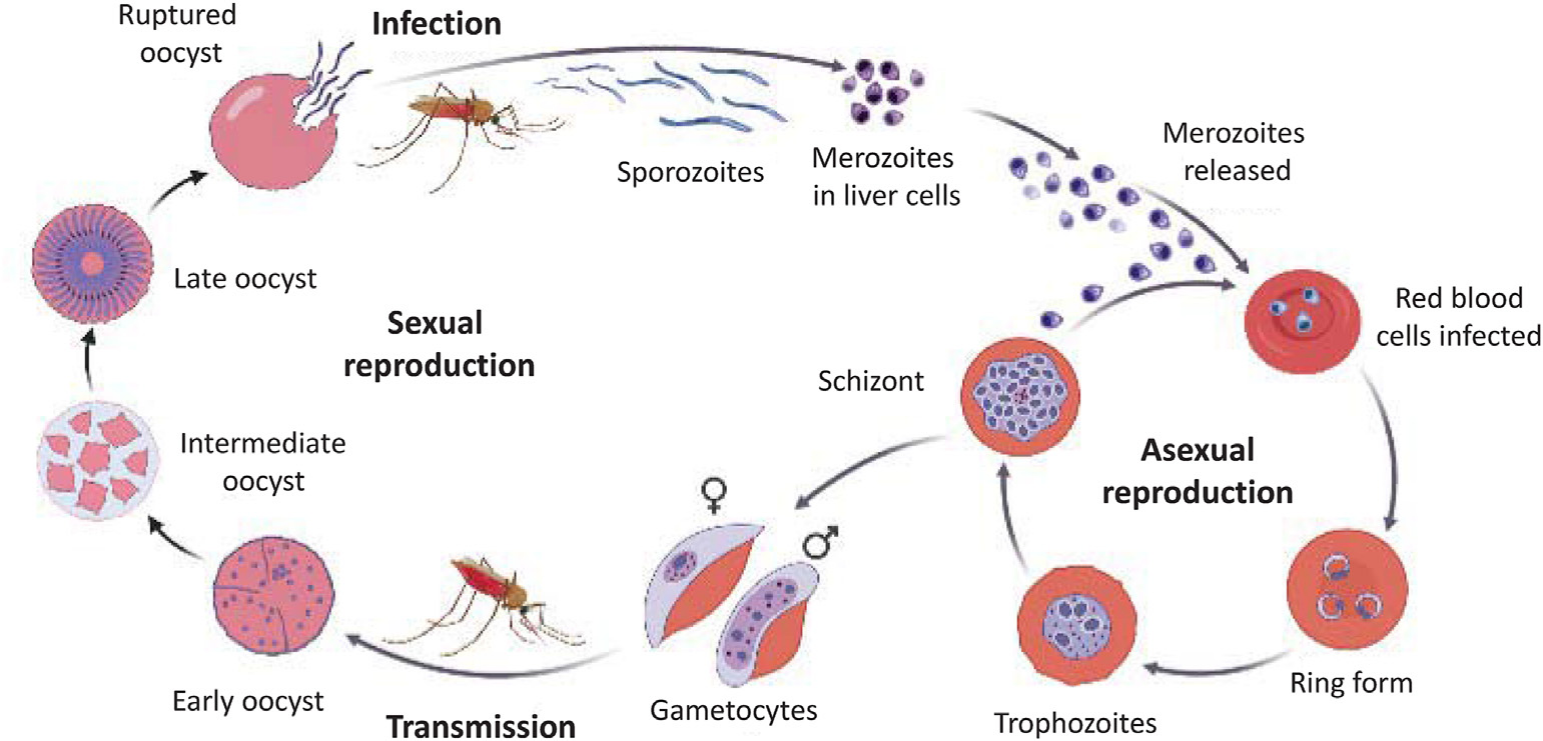
The life-cycle of *Plasmodium* spp.

The life-cycle of *Plasmodium* spp. involves two hosts. During blood-feeding, a *Plasmodium*-infected female *Anopheles* mosquito innoculates sporozoites into the bloodstream of the human host. Sporozoites reach the liver and mature into schizonts, undergo rounds of schizogony (asexual reproduction) within the hepatic cells generating merozoites, and rupture while releasing merozoites into the bloodstream (Fig. 1). Merozoites infect red blood cells (RBCs), transform into ring-stage trophozoites, which mature into schizonts and replicate manyfold; schizonts rupture and release merozoites in the bloodstream (Grüring et al., 2011). These rhythmic cycles resulting in the release of merozoites cause high fever as well as severe anaemia due to the destruction of the red blood cells. In severe cases, infected RBCs adhere to blood vessels and are being sequestered in microvascular beds throughout the body leading to microvascular obstruction. Some asexual blood forms of the parasites differentiate into male and female sexual stages (gametocytes) that are taken up by another female mosquito during blood-feeding. In the mosquito stomach, male and female gametocytes develop into zygotes, mature into ookinetes, which after penetrating the mosquito midgut wall transform into oocysts. The oocysts then grow and rupture, releasing sporozoites. The sporozoites then travel to the mosquito salivary glands and reinfect a new human host during the mosquitoʼs next blood-feeding (Grüring et al., 2011).

## 3 Microscopy methods

The traditional laboratory method for malaria diagnosis is the microscopic observation of thick and thin blood smears, and this remains the “gold standard” method (Table 1). A patient’s blood sample is taken from a finger prick or by venepuncture and prepared on two slides to make one thick and one thin blood smear. The thick smear is a droplet of blood placed on the slide, and the thin smear is a small droplet spread out across the clear area of the slide. Only thin smears may be fixed with methanol, and both are stained, most commonly with 4% Giemsa dye. They are then placed under a light microscope for observation. Thick smears are most useful for the initial diagnosis of malaria as they allow observation of a greater volume of blood and *Plasmodium* parasites may be low in density. Because these smears are not usually fixed, blood cells undergo haemolysis, leaving only parasites and leukocytes visible. Thin smears are often used for species identification and estimating parasitemia, as it is easier to observe individual *Plasmodium* parasites in this preparation (Wahab et al., 2020). The trained technicians or health professionals can also detect the morphological differences in *Plasmodium* spp., dependent on the life-cycle stage of the parasite.

**Table 1:** Microscopy methods used for the diagnosis and species identification of *Plasmodium* spp. infections, with examples of their applications, benefits and limitations.

Type	Benefits	Limitations	Uses and applications	Reference
Light microscopy	Cheap to perform; low-cost equipment; potential to diagnose other infections present	Time-consuming; relatively low sensitivity; high error rate for species identification	“Gold standard” method; technique recommended by the WHO for point-of-care patient diagnosis and speciation; used in laboratory studies	Barber et al. (2013)
Fluorescent dyes	Enhances diagnostic sensitivity	Dyes are not species-specific; some dyes have toxicity	Used to increase point-of-care diagnostic sensitivity, but do not enhance identification specificity	Guy et al. (2007)
Quantitative buffy coat (QBC)	Increases diagnostic rapidity and sensitivity for *P. falciparum*	Reduces sensitivity for non-*P. falciparum* species	Used to increase point-of-care diagnostic sensitivity	Secardin & Le Bras (1999)
Magnetic deposition	Cheap; increases sensitivity	Not species-specific; ring-stage parasites underrepresented	Used to increase point-of-care diagnostic sensitivity.	Zimmerman et al. (2006)

The sensitivity of microscopic diagnosis is dependent on the parasitemia of *Plasmodium* spp. in the blood. Many blood samples may be incorrectly identified as normal when *Plasmodium* parasites are present at a density too low to allow their detection in a single sample, meaning malaria patients may not receive treatment or treatment may be delayed (McKenzie et al., 2003). The studies into the accuracy and sensitivity of microscopic detection of *Plasmodium* spp. suggest that, while this method may be useful for general diagnosis as long as parasitaemia is high enough, it is not reliable as a means to identify infection due to a relatively high rate of human error (Barber et al., 2013). Mixed infections, with multiple species of *Plasmodium*, are often underreported, and differentiation between *Plasmodium* spp. is sometimes incorrect (Wongsrichanalai et al., 2007). This type of error can lead to the prescription of an ineffective drug course, to which the parasite is likely to be resistant, or that does not provide the required protection against relapse. The microscopic methods are a labour-intensive process, as each sample must be studied individually, and this requires a degree of expertise and experience to achieve a reliable identification of species or genus.

To combat some of these issues, methods have been developed to improve the specificity and sensitivity of this technique. For example, fluorescent dyes, such as acridine orange can be used to dye *Plasmodium* DNA within infected blood cells, to make parasites more easily detectable under a fluorescent microscope (Guy et al., 2007). The dye bound to DNA within *Plasmodium* nuclei is excited by blue light and emits a green colour, while the cytoplasm emits a yellow-orange hue. Some dyes are moderately toxic and require careful handling. The quantitative buffy coat (QBC) technique uses this principle, as well as centrifugation of samples within small capillary tubes to separate blood components. This technique enhances the diagnostic sensitivity for *P. falciparum*; however, it reduces sensitivity for the diagnosis of other *Plasmodium* spp. due to differential distribution in the capillary tube (Secardin & Le Bras, 1999). Alternatively, magnetic deposition microscopy harnesses the magnetic properties of hemozoin, a by-product of *Plasmodium* metabolism, to concentrate parasites onto the slide preparation using a magnet. This increases the sensitivity of diagnosis at low *Plasmodium* density (Zimmerman et al., 2006). Furthermore, microscopy can be semi-automated, by replacing humans in the role of species identification with trained computers. This allows a more standardised approach to morphological identification, increases capacity, and lessens the need for human expertise once the programme is adequately trained, but requires specialist equipment (Díaz et al., 2009).

## 4 Serodiagnostic assays

Serodiagnostic methods are used to recognise *Plasmodium*-specific antibodies or antigens circulating in patients blood (Table 2). The immunofluorescence antibody test (IFAT) detects *Plasmodium* species-specific both IgG and IgM antibodies mounted by the body’s immune response against the asexual blood-stage of *Plasmodium* parasites. Once a patient is infected, these antibodies are produced within two weeks and circulate in the body for 3–6 months after parasites have been cleared. The test itself is made from species-specific antigens held on a slide, that bind complementary antibodies in the serum sample. A secondary antibody with a fluorescent tag binds these to form a complex that is visible under fluorescence microscopy. This process is simple to perform and sensitive, but is time-consuming and requires the appropriate equipment and trained personnel (van den Hoogen & Drakeley, 2015). Due to the need for antibodies to develop to the parasites before these can be detected, these tests are not very useful for patient diagnosis and informing treatment in an acute infection, but have been harnessed for population surveillance, epidemiological studies, and for screening donated blood in non-endemic countries, where acquired immunity is not widespread (Seed et al., 2005; Tangpukdee et al., 2009). This technique is used for targeted screening of donated blood from individuals identified as higher risk by a survey assessment, in non-endemic regions such as France (Oh et al., 2008).

**Table 2:** Serodiagnostic assays used for the diagnosis and species identification of *Plasmodium* spp. infections, with examples of their applications, benefits and limitations.

Method	Type	Benefits	Limitations	Uses and applications	Reference
Antibody tests	Immunofluorescence antibody test (IFAT)	High sensitivity and specificity; species-specific to a degree	Time-consuming; interspecies cross-reactivity; false negatives prior to antibody development	Detects circulating anti-malaria antibodies. Useful for screening blood for epidemiological studies in non-endemic countries due to lack of acquired immunity	Oh et al. (2008); Tangpukdee et al. (2009)
	Indirect enzyme-linked immunosorbent assay (ELISA)	High throughput	Relatively low sensitivity	Detects circulating anti-malaria antibodies. Useful for screening blood for epidemiological studies	van den Hoogen & Drakeley (2015)
Antigen tests	Enzyme immunoassay (EIA) or sandwich ELISA	Direct detection of current infection; no need to wait for antibodies to develop	Relatively low sensitivity	Directly detects malaria-specific antigens. Useful for screening blood and tissue donations in endemic countries; epidemiological studies	Kitchen et al. (2004)
	Rapid diagnostic test (RDT)	Very simple, rapid, inexpensive; differentiation of *P. falciparum* and *P. vivax*	Reduced sensitivity for non-*P. falciparum* species diagnosis	Recommended by the WHO for point-of-care diagnosis. Useful for low-resource settings and field studies	Wahab et al. (2020)

The indirect enzyme-linked immunosorbent assay (ELISA) is another method used to detect *Plasmodium* species-specific antibodies. A serum sample is incubated on a 96-well plate covered with malaria-specific antigens. The enzymes catalyze the substrates resulting in a colour change indicating the presence of a *Plasmodium*-specific antibody, for example, horseradish peroxidase (HRP) and alkaline phosphatase (AP). This colour change can be quantified using a spectrophotometer. This method can be carried out for *P. falciparum* samples simultaneously with high throughput but lacks sensitivity compared with IFAT (van den Hoogen & Drakeley, 2015).

Another approach to identifying malaria infection is to directly detect parasite-specific antigens. This allows the diagnosis to be carried out for acute malaria infection. Therefore, these tests are much more useful for diagnosing and treating an acute malaria infection. Like the indirect ELISA, the enzyme immunoassay (EIA) or sandwich ELISA, uses antibodies bound to wells of a plate. The patient sample is added and *Plasmodium* species-specific antigen is captured by these primary antibodies. The plate is washed to release any unbound antigens, and an enzyme-conjugated secondary antibody is introduced. The plate is washed once more, and a substrate is added that undergoes a colour change in the presence of the enzyme attached to the secondary antibody. This can be quantified using a spectrophotometer (Noedl, 2014). This technology has been demonstrated for screening donated blood and tissue samples, where it performed comparably to IFAT, but with relatively low sensitivity for *P. vivax* infections (Kitchen et al., 2004).

Among the greatest advances in *Plasmodium* diagnosis is the rapid diagnostic test (RDT), a portable immunochromatographic lateral flow assay that gives the result of malaria infection within minutes, and is very intuitive to use and interpret. Some types of RDT can distinguish *P. falciparum* infection from other species, due to their recognition of species-specific antigens. Most RDTs work by adding a small finger-prick blood sample into a cassette strip. The antibodies complementary to the antigen of interest are conjugated with a dye, and bind the antigen in the blood sample to form a complex. A chaser buffer is added to the strip to flush the sample through the membrane and past through a line of bound antibodies that capture any antigen-antibody-dye complexes, effectively sandwiching the antigen between antibodies. If the antigen is present in sufficient amounts, this line binds enough dye to become visible to the naked eye. There is a line for the test result, and a separate control line to ensure enough sample and buffer has been added to generate this line further along the membrane (Wahab et al., 2020). The *P. falciparum*-specific tests often recognise histidine-rich protein 2 (HRP-2), which is unique to this species, or lactate dehydrogenase (LDH) proteins. The LDH proteins are present in all *Plasmodium* species, but different species-specific epitopes of the antigen can be targeted by antibodies within the assay (Bell & Visser, 2016). Other RDTs are designed as a “dipstick”, with a strip that can be added directly to a sample and allow its passage along the membrane *via* capillary action. Some RDTs detect both *P. falciparum* and *P. vivax* species-specific antigens on a separate line to allow identification within a single test. Most RDTs are relatively sensitive for *P. falciparum* infection but less sensitive for detecting non-*falciparum* species (Wongsrichanalai et al., 2007; Jimenez et al., 2017), although this is improving with newer tests (Hailu & Kebede, 2014). The challenges with this method include the discovery of *P. falciparum* isolates that show deletion of HRP-2 or HRP-3, and therefore cannot be detected by the common RDT (Gamboa et al., 2010). The performance of these tests for field use has been evaluated by many groups, and this assay is recommended by the World Health Organization in areas where traditional microscopy is not feasible (WHO, 2015).

One method of *Plasmodium* diagnosis is to detect hemozoin, an insoluble and inert crystal that is a by-product of *Plasmodium* metabolism of haemoglobin. It has recognisable optical and magnetic properties, making it a useful marker of infection. It can be detected using dark-field microscopy, and the shape or structure of its crystal formation differs between species; however, this is not generally used as a method to detect malaria infection (Noland et al., 2003). The hemozoin is detected using other optical methods such as mass spectrometry or flow cytometry (Hegg, 2014).

## 5 Molecular methods

More recent developments in malaria diagnostics have focussed on the detection and amplification of *Plasmodium* nucleic acids (Table 3). Like antigen detection, this allows the detection of *Plasmodium* spp. directly; however, because nucleic acids can be amplified from a minute amount of DNA, these methods are often far more sensitive and able to detect parasites at a much lower density. For an assay to give reliable results, high sensitivity must be accompanied by satisfactory specificity to limit the rate of false-positive results. This presents a challenge for molecular methods, as miniscule amounts of contaminating or off-target DNA can be amplified along with a sample, potentially altering results. Furthermore, many molecular techniques including polymerase chain reaction (PCR), loop-mediated isothermal amplification (LAMP) and sequencing methods allow more data to be gathered about the identification of *Plasmodium* spp. and improve the characterisation of the DNA sequences and loci of interest (Tangpukdee et al., 2009).

**Table 3:** Molecular methods used for the diagnosis and species identificaton of *Plasmodium* spp. infections, with examples of their applications, benefits and limitations.

Method	Type	Benefits	Limitations	Uses and applications	Reference
PCR-based assays	Multiplex PCR	Detects all species in a single reaction; relatively quick	Requires costly equipment and expertise	Molecular methods to detect parasite-specific DNA sequence in multiple samples simultaneously; useful in epidemiological and phylogenetic studies and for confirmation of clinical diagnosis malaria positive samples	Snounou et al. (1993); Hänscheid & Grobusch (2002); Padley et al. (2003); Boonma et al. (2007)
	Nested PCR	High level of specificity	Requires separate reaction for each *Plasmodium* species, costly equipment and expertise		
	Real-time quantitative PCR (qPCR)	Quantifies copy number and indicates parasite number	Expensive to perform and requires costly equipment and expertise		
	Ligase chain reaction (LCR)	Detects all species in a single assay	Relatively low sensitivity	Enzyme-based multiplex reaction used in conjunction with PCR; useful in large epidemiological studies	McNamara et al. (2004); Han et al. (2007); Mueller et al. (2009)
Isothermal tests	Loop-mediated isothermal amplification (LAMP)	Requires no thermocycler; rapid; easy to interpret test results	Requires expertise and complex primer design	Enzyme-based DNA amplification; useful for point-of-care diagnosis and species identification in a field setting; useful for routine blood screening	Sirichaisinthop et al. (2011)
	Nucleic acid sequence-based amplification (NASBA)	Requires no thermocycler or sample preparation; high sensitivity at low parasitaemia	Highly sensitive to temperature changes	Enzyme-based DNA amplification; useful in a reference laboratory setting for diagnosis and species identification	Marangi et al. (2009); Oriero et al. (2015)
	Thermophilic helicase-dependent amplification (tHDA)	Requires no thermocycler or sample preparation	Relatively low sensitivity at low parasite levels	Enzyme-based DNA amplification; useful for point-of-care diagnosis and species identification in a field setting	Li et al. (2013); Oriero et al. (2015)
Sequencing methods	Sanger sequencing	Provides detailed haplotype data	Slower than Ilumina; requires expensive equipment and expertise; extensive data analysis	Large data output useful for surveillance and phylogenetic studies	Doctor et al. (2016)
	Illumina MiSeq	Provides detailed haplotype data; faster than Sanger sequencing	Requires expensive equipment and expertise; extensive data analysis	Large data output useful for surveillance and phylogenetic studies	Ishengoma et al. (2019); Wahab et al. (2020)

### 5.1 PCR-based assays

PCR-based methods are based on the amplification of *Plasmodium* DNA using a thermocycler. These are among the most sensitive and specific techniques for malaria diagnosis and *Plasmodium* spp. identification. A single *Plasmodium* parasite can be detected in 1 μl of blood, which makes PCR around 50 times more sensitive than microscopy or antigen detection assay (RDT). First, DNA is extracted from the patient blood sample. The primers or short oligonucleotide strands are designed to be complementary to sections of the *Plasmodium* DNA flanking the region to be amplified. The most common genetic marker used for malaria diagnosis is often a region of the 18S rDNA that has a sequence unique to each species of *Plasmodium* and contains multiple copies. The sample and reagents are put through cycles of temperature changes, causing DNA to first denature into two strands, then these target strands bind the primers. Next, a polymerase enzyme initiates transcription of complementary strands to target strands by adding free nucleotides in the mixture. This process is repeated as the mixture is heated and cooled, amplifying the original DNA exponentially throughout the process (Murphy, 2017). The whole process can be automated and many samples run concurrently. The results are visualised using gel electrophoresis to separate any amplified DNA products by size, to determine if the expected size of the fragment has successfully been amplified, or by allowing a probe to hybridise with the target DNA sequence, such as in a southern blot. Mixed malaria infections, and infections with very low parasitaemia, can be detected much more easily due to the high sensitivity and specificity of this PCR assay (Johnston et al., 2006). Despite the many benefits of PCR over non-molecular methods, it does require specialist equipment and expertise, making it less practical for diagnosis in the field in endemic regions (Tangpukdee et al., 2009). Furthermore, since PCR requires pre-designed primers, prior knowledge of the sequence upstream of the genetic region of interest is necessary. For primers to work well, they are designed according to a set of criteria, reducing the flexibility of the exact PCR product that can be generated (Dieffenbach et al., 1993).

Several types of PCR can be used for malaria diagnosis and *Plasmodium* identification, each with its relative benefits and drawbacks. For example, multiplex PCR uses multiple sets of primers in a single PCR reaction to target several fragments of DNA sequence and generates products of varying corresponding sizes. The thermocycling conditions must be adapted to work with all primers simultaneously. These products can be visualised by separating them by size using gel electrophoresis. Multiplex PCR assays developed for malaria diagnosis are highly specific for each species of *Plasmodium* (Padley et al., 2003; Lau et al., 2015). Nested PCR uses two sets of primers in two separate PCR reactions, to create a more pure product. The initial PCR reaction generates a product which is then used in a second PCR reaction along with primers that are partially different from the first set, designed to amplify a region within the initial product. This increases specificity by purifying the target product from the first PCR reaction. The nested PCR assay developed by Snounou et al. (1993) is still a widely used method for *Plasmodium* identification. The real-time quantitative PCR (qPCR) is a technique used to quantify the amount of target sequence present in an initial *Plasmodium*-positive blood sample, thereby indicating the level of parasitaemia present in the blood, for example, this is done by including a fluorescent dye, or a fluorophore with a nucleic acid probe, in the PCR reaction. The starting nucleic acid quantity is calculated from the number of cycles of amplification needed to reach a cycle threshold above background fluorescence, using melting curve analysis (Wanger et al., 2017). The assays developed for the identification of all species of *Plasmodium* in multiplex using real-time PCR have performed well with clinical samples (Mangold et al., 2005).

Boonma et al. (2007) compared these three PCR-based techniques for diagnosis and identification of field samples with *P. falciparum*, *P. vivax* and mixed infections measured with the microscopy “gold-standard” method. They found that real-time PCR was the only 100% sensitive and specific technique for diagnosis of each species and mixed infections and the quickest molecular method. All three molecular techniques outperformed traditional microscopic methods, which had a false-negative rate of 9.6% (Boonma et al., 2007). PCR-based methods are still less commonly used for point-of-care malaria diagnosis and *Plasmodium* spp. identification due to the delay in providing results but are often used in disease surveillance and epidemiological studies, using dried blood spot samples (Singh et al., 1999; Hänscheid & Grobusch, 2002).

Ligase chain reaction (LCR) is a multiplex DNA amplification reaction used to diagnose and identify *Plasmodium* spp. that can be used in conjunction with PCR. The method uses a thermostable DNA ligase enzyme and two adjacent sets of forwarding and complementary primers. A gap of 1–3 base pairs between the sets of primers provides a template for the DNA ligase to bind, and due to its highly specific action will not bind if any mismatches are present in the sequence. A thermostable DNA polymerase is used to amplify successfully ligated sequences. LCR can be, therefore, used for simultaneous diagnosis of all *Plasmodium* spp. in a single assay, with high sensitivity and specificity (McNamara et al., 2004). This has been used for epidemiological surveillance of multiple *Plasmodium* spp. in Papua New Guinea with far higher sensitivity than traditional light microscopy (Mueller et al., 2009).

### 5.2 Isothermal tests

Loop-mediated isothermal amplification (LAMP) of DNA is a technique for amplifying *Plasmodium* spp. nucleic acid that does not require thermocycling equipment. This is due to its utilisation of a bacterial DNA polymerase that has a high displacement activity, meaning it can displace complementary strands to begin transcription of another, negating the need for temperature-dependent cycles of denaturation and annealing. Two or three sets of primers are used to bind six target sites of the *Plasmodium* genetic marker. The DNA polymerase begins transcription at two inner primer sites, synthesising complementary strands. Next, transcription begins at two outer primer sites, displacing the first complementary strands, which form loops and together create a dumbbell structure. This structure, with the assistance of two-loop primers, allows successive displacement and transcription, producing many copies of the DNA sequence. A positive result can be detected by eye or using machinery, due to the build-up of insoluble by-products of the reaction. This may be visible as a precipitate of white magnesium pyrophosphate, for example, or by using fluorophores such as calcein that becomes visible upon binding the by-product. Alternatively, positive results can be detected by measuring the change in turbidity of the solution (Polley & González, 2014). The LAMP tests can be carried out in around 30 min, run in a simple water bath, and have similar sensitivity and greater specificity than light microscopy, making this an attractive option for routine screening of blood samples in endemic areas (Han et al., 2007) or point-of-care diagnosis and speciation in the field (Sirichaisinthop et al., 2011).

Nucleic acid sequence-based amplification (NASBA) uses three enzymes, including a reverse transcription and strand displacement polymerase, to amplify a target sequence of *Plasmodium* without a thermocycler. It can be used for diagnosis and species identification and carries less risk of contamination than PCR due to its lack of a DNA denaturation step. The reaction cannot be controlled by the number of heat cycles as PCR and its active enzymes denature at temperatures above 41 °C (Oriero et al., 2015). This has been used for screening migrants moving from endemic areas of sub-Saharan Africa to Europe (Marangi et al., 2009).

Thermophilic helicase-dependent amplification (tHDA) uses DNA helicase to separate the DNA strands, and the DNA is coated with DNA-binding proteins. The primers bind adjacent to the target DNA, and a DNA polymerase extends the sequence from the primers. The synthesized double-stranded DNA is then separated by a helicase and the next round of amplification begins (Oriero et al., 2015). This platform is cheaper to run than PCR because it does not require thermocycling, and has performed well for diagnosis of malaria infections and *Plasmodium* spp. identification using human blood samples without prior preparation or DNA extraction, making it a relatively practical option for field use. This assay was validated for clinical use by using it for malaria diagnosis and *Plasmodium* spp. identification in patient samples in the USA (Li et al., 2013).

### 5.3 Sequencing methods

Traditionally, Sanger sequencing, also known as chain termination sequencing, has been used to diagnose infections following PCR amplification of *Plasmodium* parasite DNA. The method applies dideoxynucleotides (ddNTPs) labelled with fluorescent tags to terminate DNA duplication through arbitrary integration into the sequence. The separated sequences through capillary electrophoresis pass through a laser light capable of distinguishing between the fluorescent tags matching to the base pairs at the time of termination, which will convert the sequence to identify species through computer software. Sanger sequencing was used to identify patients with mixed-species infections and non-*P. falciparum* mono-infections for surveillance in the Democratic Republic of the Congo. This technique was chosen due to the lack of sensitivity of non-*P. falciparum* diagnoses of RDTs (Doctor et al., 2016).

The Illumina MiSeq can be extremely useful for diagnosis of infection and *Plasmodium* spp. identification for surveillance applications, where large numbers of samples are run with fewer time constraints. A high-throughput deep amplicon sequencing of *P. falciparum* and *P. vivax* using the Illumina MiSeq platform has recently been developed and validated in endemic regions (Ishengoma et al., 2019; Wahab et al., 2020).

## 6 Other applications of the sequencing method

The data generated by sequencing methods can be used to study the disease epidemiology of *Plasmodium* spp. For example, the multiplicity of *Plasmodium* infection (MOI) refers to the number of genetically distinct parasite genotypes in a single infection and is important for many genetic analyses. It is usually calculated by genotyping of the desired genetic markers using sequencing methods; however, this often underestimates the number of genotypes present in a single infection. Deep sequencing is much more sensitive and able to pick up low copy numbers of unique genotypes. This method has shown that multiple infections are common in regions of high transmission (Assefa et al., 2014). This means that data generated by high-throughput sequencing can also inform us about the nature of disease dynamic and transmission, therefore be used for vector and malaria control strategies.

Sequencing methods may be harnessed to generate parasite genotype data to inform the likely clinical presentation of infection, by distinguishing recrudescence of an existing infection from reinfection by new *Plasmodium* parasites. This is done by genotyping the highly polymorphic genes (glutamate rich protein (*glurp*), merozoite surface protein 1 (*msp1*) and merozoite surface protein 2 (*msp2*) genes) to create an infection profile, that can be compared between initial and recurring infections. This is less time-effective in areas of high transmission, where there is high MOI, and therefore multiple *Plasmodium* parasites in a single infection which could lead to mistaking recrudescence for a new infection (Ishengoma et al., 2019). This distinction between recrudescence and reinfection has important implications for the treatment of patients, as it may indicate whether a drug has effectively cleared the infection or not.

## 7 Identification of *Plasmodium* spp. in a nutshell

For the point-of-care malaria diagnosis and *Plasmodium* spp. identification, microscopy is still considered the “gold standard”; however, this technique has significant limitations. In resource-poor malaria-endemic regions, the RDT is a great option due to their ease of use and fast results. They are relatively stable in storage and transport with minimal equipment required. The RDT is very useful for the diagnosis of *P. falciparum*, and more recent tests have shown sufficient sensitivity for the diagnosis of *P. vivax*. These tests tend to give a suboptimal performance for the diagnosis of *P. ovale*, *P. malariae* and *P. knowlesi* (Yerlikaya et al., 2018). The RDT has become invaluable in recent years for point-of-care diagnosis in *P. falciparum-*endemic regions, but in areas where mixed infection is possible, and where resources are available, PCR-based methods may be more appropriate (Hawkes & Kain, 2007).

For screening of blood samples, such as those from refugees from endemic regions entering a non-endemic region, PCR may be most useful where available resources are sufficient. The PCR allows *Plasmodium* spp. identification that may also inform treatment guidance. For example, in a study of refugees entering Canada and Pakistan, microscopy and RDT performed very poorly against PCR. All malaria-positive PCR results came from asymptomatic people, highlighting the need for high sensitivity in such screening tests as a clinical diagnosis is not possible (Ndao et al., 2004; Wahab et al., 2020). Where resources are limited, RDT may be used instead. The screening of donated blood samples in endemic areas would be incredibly useful, as current restrictions placed on higher-risk groups on making donations likely prevent too many people from donating, contributing to shortages in the blood supply. This would, however, require a very low-cost method with a rapid turnout of results in addition to high specificity and sensitivity and the perfect method for this does not exist (Erdman & Kain, 2008). Currently, some countries implement serological testing of higher-risk blood donors following a 4–6-month deferral period to allow them to donate earlier than the normal 1+ year deferral period would allow, if their serology test is negative. This has increased the availability of thousands of units of blood annually but does potentially lead to exclusion of some donors that have persistent antibodies from a past infection that has been cleared (Seed et al., 2005). Alternatively, there is some evidence to suggest that using a questionnaire to identify higher-risk donors and running PCR diagnosis on these samples is more cost-effective than relying on questionnaires alone, due to the increased inclusion of negative samples (Shehata et al., 2004).

For the surveillance of malaria, there is often less urgency for rapid turnaround of results and a greater need for high throughput capacity. In this case, the samples can be sent to larger laboratories with more advanced and expensive equipment, and more specific expertise, than may be available in smaller facilities. The cost per sample should still be kept minimal (Erdman & Kain, 2008). In endemic areas, passive surveillance is often insufficient alone due to its reliance on clinical diagnosis, and therefore symptomatic disease, and also due to limited access to healthcare facilities and the subsequent tendency for people to self-treat. Active surveillance often relies on diagnosis by microscopy, which has low sensitivity. The PCR-based methods are more effective than microscopy and are increasingly utilised in larger laboratories that can receive blood samples from smaller facilities. The LAMP has similar performance rates to PCR for diagnosis of *P. falciparum*, and perhaps more practical in low-resource endemic settings (Tambo et al., 2018). The high-throughput deep amplicon sequencing of *P. falciparum* and *P. vivax* using Illumina MiSeq Platform was recently developed and validated the sensitivity of this assay in the endemic regions (Wahab et al., 2020).

## 8 Conclusions

The fight against malaria has been challenging, with many setbacks and hurdles. Furthermore, antimalarial drug resistance has generated a huge threat to healthcare. Continuous innovation in technological development has helped the scientific community to develop novel tools to improve the understanding for the control of malaria including its most severe forms. To this end, in modern methods, many drawbacks still need to be improved. Therefore, the assay of choice must depend on its practicality and the desired application for its results. The development of cheaper, simpler and faster methods used for malaria diagnosis and *Plasmodium* spp. identification means patients have a better chance of receiving appropriate treatment while minimising the selection of parasite drug resistance traits. More sensitive, specific, automated and high-throughput methods for diagnosis have greatly boosted the capacity for surveillance of the disease. To conclude, these along with such other features must contribute to improved time-bound and effective treatment for patients suffering from malaria to decrease its burden in endemic countries.

## Funding

The study was supported by the Roslin Institute facilities funded by the Biotechnology and Biological Sciences Research Council (BBSRC).

## **CRediT** author statement

Umer Chaudhry, Lucinda Slater, Neil Sargison: conceptualization, data curation, formal analysis, methodology, resources, writing - original draft, writing - review & editing. Shoaib Ashraf, Osama Zahid, Qasim Ali, Muhammad Oneeb, Muhammad Haroon Akbar, Muhammad Ilyas Riaz, Kiran Afshan: resources, writing - review & editing. All authors read and approved the final manuscript.

## Declaration of competing interests

The authors declare that they have no known competing financial interests or personal relationships that could have appeared to influence the work reported in this paper.
